# Multifunctional Nanoparticles and Nanopesticides in Agricultural Application

**DOI:** 10.3390/nano13071255

**Published:** 2023-04-02

**Authors:** Jiaming Yin, Xiaofeng Su, Shuo Yan, Jie Shen

**Affiliations:** 1 Department of Plant Biosecurity and MARA Key Laboratory of Surveillance and Management for Plant Quarantine Pests, College of Plant Protection, China Agricultural University, Beijing 100193, China; bh2022136@cau.edu.cn; 2 College of Plant Protection, Sanya Institute of China Agricultural University, Sanya 572025, China; 3 Biotechnology Research Institute, Chinese Academy of Agricultural Sciences, Beijing 100081, China; suxiaofeng@caas.cn

**Keywords:** nanoparticles, nano delivery system, nanopesticide, pest management, sustainable agriculture

## Abstract

The unscientific application of pesticides can easily cause a series of ecological environmental safety issues, which seriously restrict the sustainable development of modern agriculture. The great progress in nanotechnology has allowed the continuous development of plant protection strategies. The nanonization and delivery of pesticides offer many advantages, including their greater absorption and conduction by plants, improved efficacy, reduced dosage, delayed resistance, reduced residues, and protection from natural enemies and beneficial insects. In this review, we focus on the recent advances in multifunctional nanoparticles and nanopesticides. The definition of nanopesticides, the types of nanoparticles used in agriculture and their specific synergistic mechanisms are introduced, their safety is evaluated, and their future application prospects, about which the public is concerned, are examined.

## 1. Introduction

In recent years, crop diseases and insect pests have caused substantial economic losses to agricultural production throughout the world. Pesticides play a pivotal role in the prevention and control of pests and diseases [1,2]. However, there is an urgent requirement for the scientific application of pesticides to green food production to reduce the number of chemical agents used to improve the efficiency of disease and pest prevention and control, and to control pest resistance. To increase the efficiency of pesticides, more-efficient pest control technologies must be developed, consistent with green and sustainable agricultural development [3].

Nanotechnology has a wide range of potential applications, which will revolutionize the fields of science and technology in the 21st century, including in the sectors of materials science, biomedicine, agriculture, and industry, and will play important roles in various changing fields [4]. The application of nanotechnology to plant science and agriculture is one of the fastest-growing areas of nanoresearch [5,6,7,8,9,10,11]. In agriculture, nanoparticles are mainly used to effectively deliver exogenous insecticidal factors to improve plant health, expand the insecticidal spectrum, reduce pesticide use, prolong the period of efficacy, and reduce environmental pollution. Nanoparticles can be used as adjuvants to assist in the delivery of chemical or botanical pesticides, which can both improve the efficacy of pesticides and reduce the amount of pesticide applied [12,13]. As well as acting as adjuvants for chemical or botanical pesticides, nanoparticles can be used to deliver biological pesticides such as double-stranded RNAs and toxic *Bacillus thuringiensis* (Bt) proteins, with broad potential utility in pest control [14,15,16].

Although the use of nanoparticles in agriculture has been growing exponentially, few reviews have examined all aspects of nanopesticides and nanoparticles. In particular, emerging nanoparticles and their synergistic mechanisms have been overlooked. In this paper, we discuss various aspects of the preparation of nanopesticides, their synergistic mechanisms and safety, and their frontier applications; summarize the research status of nanoparticles and nanopesticides in agricultural production; and clarify the important roles and potential utility of nanopesticides in the development of modern agriculture.

## 2. Nanopesticide Definition

Nanopesticides are compounds that kill insects, bacteria, and weeds, and are prepared at the nanometer scale with physical, physicochemical, and chemical methods. At present, the classification of nanopesticides is predominantly based on particle size, and there is no uniform globally accepted definition. Some studies have defined nanopesticides as having an upper size limit of 100 nm, which is too simple and excludes many nanopesticides [17]. Kah and Hofmann defined nanopesticides broadly as plant-protection products with a particle size of <1000 nm, which are prefixed with “nano”, or products with new characteristics related to their small particle size. Therefore, in a broad sense, nanopesticides are not limited to a size of 100 nm [18]. The category includes not only pesticides, fungicides, herbicides, and rodenticides, but also active substances, such as plant attractants and plant-growth regulators, that can improve plant resistance to pests and diseases. The small size of nanoparticles can improve the saturation and solubility of the pesticides in water, thus improving their stability and dispersibility in water for enhanced control efficacy and utilization rates [19,20].

Some nanomaterials have specific antibacterial or insecticidal properties, and can be used directly as the active components of pesticides [21]. For instance, silver nanoparticles controlled early tomato blight, and increased the fresh weight and chlorophyll content of tomatoes by 32.58% and 23.52%, respectively [22]. The application of magnesium oxide nanoparticles significantly inhibited *Ralstonia solanacearum*, the bacterial wilt pathogen of tomato, and reduced the disease index [23]. Chitosan can be used as a plant elicitor to activate the defense responses of crops, playing an important role in disease control [24,25]. Copper oxide and calcium oxide nanoparticles can be used to poison *Spodoptera littoralis* [26]. The median lethal concentration (LC_50_) of copper oxide nanoparticles is 232.75 mg/L, and the LC_50_ of calcium oxide nanoparticles is 129.03 mg/L.

Nanomaterials also have many excellent physical and chemical properties, including their small size, large specific surface area, and strong adsorption capacity, which allow them to be used as efficient carriers of pesticides. On the one hand, nanocarriers can improve the chemical stability and water dispersibility of pesticides, and on the other hand, protect the effective ingredients from degradation by high temperature and ultraviolet radiation in nature, thus promoting their efficacy [1,27]. With these advantages, researchers have developed a series of nanocarriers rich in biocompatible functional groups, which not only allow the nanonization of the pesticides and play a protective role, but also promote the rapid and efficient delivery of pesticides to target crops and pests through their active endocytosis by cells. This increases the efficacy of the pesticides, reduces the amount applied, and ensures environmental protection [15,28]. In the last decade, nanopesticide preparations based on nanoparticles have become a research hotspot. It is anticipated that the intelligent release and efficient targeted delivery of active substances can be controlled, while the amount of pesticide is limited and its potential harm restricted. The developmental direction of new pesticide nanocarriers is towards their easy degradation, minimal pollution, low price, and modification.

## 3. Nanopesticides Prepared as Inorganic Nanoparticles

Nanoparticles can not only load pesticides, but also ensure their biodegradation, environmental safety, processability, and chemical compatibility with the loaded pesticide [29]. The development of nanoparticles has provided many options. Current nanoparticles are mainly divided into two categories: inorganic nanomaterials and organic nanomaterials. Inorganic nanomaterials predominantly include silicon dioxide, metal organic skeletons, graphene, clay minerals, etc. With continuous research in recent years, a number of nanopesticides with inorganic nanocarriers have been developed (Table 1).

### 3.1. Mesoporous Silica Nanoparticles (MSNs)

MSNs are characterized by a large surface area, tuning pores, modified interfaces, biocompatibility, environmental friendliness, and good adsorption performance. Since Kresge first reported MSNs, research into their preparation has advanced greatly [30]. MSNs loaded with various pesticides, including avermectin, pyromamine, hepazole, amino acid, and prochloraz, have been developed.

Pesticide-loaded MSNs can be prepared by the physical adsorption method, and these are most easily absorbed and transported by plants. The surface of the MSN contains hydroxyl and unsaturated keys, to which activated groups can be introduced through silicide coupling, branch aggregation, etc. to confer new performance capabilities on MSNs. Modified MSNs can be continuously released into the release medium, and prevent the degradation of the active ingredients by light [31,32,33,34].

### 3.2. Graphene Oxide (GO)

Graphene oxide is a two-dimensional nano-inorganic carbon material composed of single-layer carbon atoms. It has a large surface area and high thermal conductivity [35]. GO combines with newly introduced groups through covalent bonds to improve and enhance its properties. It also accumulates through π-π interactions, hydrophobic effects, and hydrogen bonds, and has great potential utility in the field of agriculture [36,37,38].

GO has been used to construct a nano-AVM-GO system with a high loading capacity, which has the release performance of pH response, which improves the anti-optical solution performance of AVM [39]. The sharp layered structure of graphene is also effective in the prevention and control of the Asian corn cricket. GO causes physical damage to the insect body wall and achieves coordinated efficiency. A nanodelivery system loaded with β-cyfluthrin and imidacloprid via GO has greater contact toxicity against insect pests because modified GO enhances the adhesion of the pesticide to the target [40].

### 3.3. Metal–Organic Frameworks (MOFs)

Metal–organic frameworks are porous crystalline materials with metal ions or clusters as the central atom and one or more organic ligands, constructed with the coordination mode, which form infinite periodic networks. The unique advantages of MOFs, such as their large surface areas, adjustable apertures, and diverse structures, have made them widely applicable in many fields [41,42,43]. The composition of MOF materials determines their flexible diversity of organic ligands and metal ions, although the types of organic ligands and metal ions used and the methods of their connection are highly specific. Therefore, MOF materials have highly diverse structures and properties. Highly stable carboxylic acid ligands are often used as organic ligands in the synthesis of MOF materials. The range of metal centers used includes almost all metal elements, including both transition elements and lanthanide metals. Zinc, iron, and copper are commonly and widely used [44]. In recent years, MOFs have been used to control the release of pesticides, which has become a research hotspot.

The modification of MOFs can enhance their properties [45]. The loading capacity of carboxymethyl-chitosan-modified MOFs for dinotefuran is as high as 24.5%, the effective components are released continuously into the release medium, and their antiphotolysis activity is 3.4 times higher than that of the original pesticide. This drug delivery system is also pH sensitive [46]. Another pesticide-loaded pH-sensitive MOF system also showed the rapid release of dopamine-modified MOFs in an acidic medium [47].

### 3.4. Clay Minerals

Clay minerals are water-bearing silicates or aluminosilicates, mainly including kaolinite, montmorillonite, attapulgite, diatomite, hydrotalcite, etc. Most clay minerals are crystalline. They have a high specific surface area that can be modified with inorganic or organic cations. These modified derivatives are excellent adsorbents for many organic compounds, the release of which into the environment can be controlled by their adsorption of active components [48,49]. Li et al. prepared a controlled-release gel loaded with the herbicide acetochlor using carboxymethyl cellulose gel and different types of clay as the raw materials [50]. The release rate was affected by the preparation conditions. Increasing the crosslinking time of the gel slowed the release of the active ingredient after it was added to the gel. The duration of acetochlor release also increased after it entered the clay. In the presence of cyclodextrin, iron-based clay can increase the adsorption of herbicide. In this slow-release system, the rate of herbicide release into sandy soil was slower than that of the ordinary preparation [51].

### 3.5. Other Inorganic Materials

The porous structure, widespread sources, and easy availability of biochar have allowed its development as a nanoparticle for pesticides. Cai et al. prepared biochar and biosilicon from straw, and loaded it with chlorpyrifos, to improve the deposition and adhesion of the insecticide on leaves and to reduce the migration of the pesticide in the soil [52]. When mesoporous selenium was loaded with thiophanate-methyl, the thiophanate-methyl in the drug-loaded system showed good water solubility and a significantly improved antifungal effect. At the same time, the drug-loaded system improved the efficiency of photosynthesis in plants, and promoted growth of plants [53]. A delivery system for PCM-SS/PMT was prepared with porous calcium carbonate microspheres (PCMs) loaded with PMT and was characterized by the slow release and effective management of the herbicide, which had a higher utilization rate and a better herbicide effect than the traditional herbicide [54]. Another drug delivery system prepared with silver nanoparticles not only displayed insecticidal activity, but also antibacterial activity, attributable to the silver nanoparticles [55].

As a widely used nonsiliceous material, mesoporous alumina has received widespread attention and has been widely applied [56,57]. Alumina has attracted researchers’ attention due to its strong antibacterial activity, its nontoxic properties, its physicochemical properties, and its potential biological applications. Alumina nanoparticles have been used for agricultural applications such as controlling tomato root rot.

**Table 1:** Nanopesticides prepared as inorganic nanoparticles.

Material	Pesticide	Target	Performance	Reference
MSN	Mildamine	Cucumber	Enhanced uptake by cucumber	[58]
PRO@DMON–GA–Fe(III)	Prochloraz	Rice	Better fungicidal activity against *Magnaporthe oryzae* with longer duration	[59]
Pro@HMS–TA–Cu	Prochloraz	Oilseed rape leaves	Better antifungal activity against *Sclerotinia sclerotiorum* and lower toxicity against zebrafish compared with prochloraz technical	[60]
Nano-AVM–GO	Avermectin	Diamondback moth	Better insecticidal effect	[39]
MOFs	Cyfluthrin	-	Slow-release performance	
Fe(III)-MOFs	Azoxystrobin	Wheat seed	Increased weight of the aboveground parts of wheat	[61]
IV-Porphyrin-MOFs	Tebuconazole	Pathogenic microbes	Good fungicidal effect	[62]
AM@CuBTC	Avermectin	Pine wilt disease	Improved solubility, photolysis performance, and pesticide efficacy	[63]
ZIF-90-KSM	Kasugamycin	Rice	Great potential synergistic antifungal functions and provides an eco-friendly approach to managing rice diseases	[64]
PRCRC	Chlorpyrifos	Tick	Excellent pH sensitivity and excellent insecticidal performance	[65]
MSN	Zobactamide	*Phomopsis asparagi*	Good bacteriostatic effect	[66]
Biochar	Glyphosate	Weeds	Good control of weeds	[67]
ZuO	Chunleimycin	-	Excellent UV blocking	[68]
GO	Pyridaben Chlorpyrifos Cypermethrin	-	Improves insecticide efficiency	[69]
GO-MSN_10_	Camptothecin	-	Good slow-release performance	[70]
PCM-SS/PMT	Prometryn	Grass	Effective control and improved utilization rate	[54]

## 4. Nanopesticides Prepared with Organic Materials

Depending on their source, organic materials can be divided into natural polymers and synthetic organic materials. Natural polymer materials occur widely in nature, and have the advantages of excellent biodegradability, good environmental compatibility, and inexpensiveness. Synthetic organic materials have the advantages of stability and plasticity. After continuous exploration and optimization in recent years, a group of synthetic organic materials characterized by environmental friendliness and biocompatibility have also been developed as nanoparticles (Table 2).

### 4.1. Natural Polymers

Chitosan is a kind of natural polymer obtained by the deacetylation of chitin, which displays good biocompatibility and biodegradability. Its NH_2_ and OH functional groups allow chitosan to react with glutaraldehyde, vanillin, and ginpinil in the preparation of microcapsules or hydrogels. These can then be combined with pesticides to prepare nanopesticides [71]. Chitosan itself can also be used as bacteriostatic material to control plant diseases [24]. Chitosan-loaded paraquat prolongs the persistence of paraquat and reduces its toxicity [72]. Drug-loaded chitosan microcapsules can protect photosensitive pesticides from photodegradation. Liang et al. prepared chitosan nanoparticles loaded with avermectin with the ion-crosslinking method. The microcapsules displayed good slow release, which effectively improved the period of retention of avermectin and enhanced its photostability [73].

Sodium alginate is a natural anionic polymeric polysaccharide that can be cross-linked with polyvalent metal cations. A large number of amino and carboxyl groups on the surface of sodium alginate can also be modified to prepare drug carriers with different physicochemical properties. Calcium alginate hydrogels loaded with *Lentinus edodes* were prepared from sodium alginate. Release from the hydrogels showed good environmental sensitivity, and the release rate of *L. edodes* increased at elevated pH, temperatures, and Na^+^ concentrations. The hydrogel continuously induced plant resistance to the tobacco mosaic virus and promoted plant growth, while having no adverse effects on aquatic zebrafish [74].

β-Cyclodextrin is characterized by its of biocompatibility and biodegradability. It has a hydrophilic external structure and a hydrophobic internal cavity [75]. Hydrophobic drugs can be incorporated into the internal cavity to form a water-soluble inclusion compound, allowing their slow release and solubilization [76,77]. Hydrophilic nanohydrogels can be prepared with the saturated solution method. β-Cyclodextrin has enhanced the water solubility and stability of DEET and capsaicin. Cellulose, starch, guar gum, and gelatin are also commonly used as carriers of pesticides, including abamectin and thiamethoxam [78].

### 4.2. Synthetic Organic Materials

Compared with natural polymers, synthetic polymers have obvious advantages as pesticide carriers [79]. First, they have great physical and chemical stability, acid resistance, alkali resistance, and erosion resistance. Second, the type and quantity of the surface functional groups can be adjusted according to the actual requirement, which allows highly targeted and flexible applications. Third, carriers with special functions can be selected according to the characteristics of the environment in which they are used [80]. Polyacrylamide and polyvinyl alcohol were used as gel encapsulation agents in the early phase of synthetic polymer research, and in recent years, the application of a polylactic acid–glycolic acid copolymer and a polyethylene glycol copolymer has been reported. A poly (lactic acid)–glycolic acid copolymer was randomly polymerized from lactic acid and glycolic acid, and has good biodegradation, encapsulation, and film-forming properties. After modification, the encapsulation rate was higher, and the slow-release performance was better [81].

Many synthetic polymers used as pharmaceutical carriers have been reported. Yang et al. used a star cationic polymer (SPc) to load abamectin through hydrogen bonding and intermolecular van der Waals forces, forming nanoscale pesticide particles with enhanced virulence against green peach aphids [82]. They also used the SPc-based nanodelivery system to deliver cyanobenamide (CNAP), and evaluated its selective toxicity against the pest western flower thrips (WFT) and the predator *Orius sauteri*. SPc-loaded CNAP showed greater selective toxicity, which enhanced the negative effects of CNAP on predators [83].

**Table 2:** Nanopesticides prepared from organic materials.

Material	Pesticide	Target	Performance	Reference
AL/PEG–acetamiprid	Acetamiprid	*Xanthogaleruca luteola*	Increased insecticidal performance	[84]
ABA@AL-CTAB	Abscisic acid	Rice	Slow-release performance and resistance to photolysis	[85,86]
SPc–calcium glycine	Calcium glycine	Tomato mosaic virus	Improved control of virus	[87]
Polyhydroxybutyrate–trifluralin	Trifluralin	Barnyard grass	Improved photostability and herbicidal activity	[88]
Chitosan–polylactic acid	Chlorpyrifos	-	Good slow-release effect	[89]
Chitosan–sodium tripolyphosphate	Hexazole alcohol	-	Good bacteriostatic effect	[90]
Beeswax–corn oil–liposomes	Deltamethrin		Resistance to photolysis	[91]
AL-azo-H@AVM	Avermectin	-	Excellent UV-blocking and controlled-release performance	[92]
AVM@P-Zein	Avermectin	-	Excellent UV-blocking and controlled-release performance	[93]
SPc–dinotefuran	Dinotefuran	Aphids	Better distribution and enhanced uptake	[94]
SPc–chitosan	Chitosan	*Phytophthora infestans*	Enhanced control effect	[95]
Polyhydroxyalkanoate	Chlorhexine	-	Greater herbicidal activity	[96]

## 5. Synergistic Mechanisms of Nanopesticides and Nanocarriers

### 5.1. Nanocarrier Increases the Contact Area between Pesticide and Target Pest

At present, most available pesticides are emulsifiable oils or wettable powders, with attendant problems, such as poor water dispersibility, low biological activity, and ineffective utilization. The poor water solubility of the effective components of pesticides is an important factor restricting the effective utilization of pesticides. Due to their small particle size, nanopesticides can improve the water dispersion of pesticides, expand the contact area between the pesticide and the target pest, and improve the bioavailability of the pesticide. Yan et al. used a star polycation (SPc) as a matrine nanocarrier. After complexation with matrine pellets, the particle size of matrine in an aqueous solution decreased from 858.38 nm to 9.12 nm, which increased its toxicity against the *Drosophila* S2 cell line and *Myzus persicae* by about 20% [28]. To confirm that SPc can be used as a general pesticide assistant, Yan et al. combined the nanocarrier with the chemical pesticide thiamethoxam. The particle size of thiamethoxam was reduced from 576 nm to 116 nm, and its stomach toxicity and contact toxicity against green peach aphids were both significantly increased by about 20% [15]. Wang et al. found that chitosan could combine with SPc, reducing its particle size from 144.61 to 17.4 nm [95].

### 5.2. Nanocarrier Promotes the Plant Uptake of Pesticide

The small size and surface modifiability of nanopesticides can promote the absorption and transport of pesticides by plants, and thus enhance the plant uptake of pesticides, especially hydrophobic chemicals. Pesticides mainly enter the plants through either foliage spray or root application. Nanoparticles are deposited on or adsorbed by the leaves, stems, and roots of plants, penetrate the cuticle and epidermis, migrate to the vascular tissues through plastids or exoplasts, and are then transported to various parts of the plants through the vascular tissues [97,98]. Dendritic macromolecular polymer nanocarriers with perylene imide can self-assemble with fluorescent nuclei into complexes that can quickly penetrate the cell wall of the plant root cap and enter the plant cell. They can also penetrate the intestinal peritrophic membrane, cell membrane, and even the body wall of pests, and enter various tissues and cells of insects [99,100]. An analysis of the internal plant uptake of nanothiacloprid and its monomer showed that the plant absorbed 1.69–1.84 times more nanothiacloprid than thiacloprid, and a plant root feeding test showed that the gastric toxicity of thiamethoxam nanopharmaceuticals against green peach aphids was about 20% higher than that of thiamethoxam [15]. Zhao et al. used mesoporous silica as the carrier of spirochetes, and the loaded spirochetes showed better deposition, absorption, and transportation on cucumber plants [101].

### 5.3. Nanopesticide Shows Increased Adhesion to Leaves

**Figure 1:**
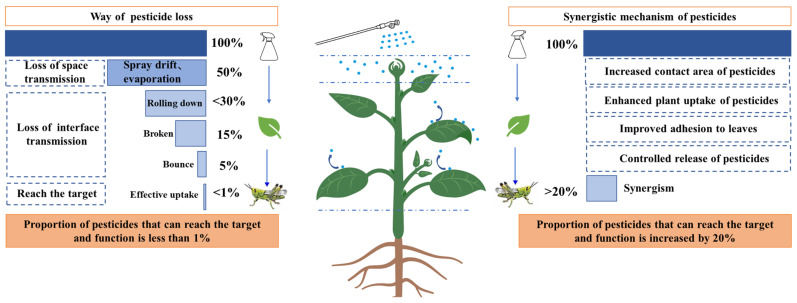
Schematic diagram of pathways of pesticide loss and synergistic approaches to improving pesticide utilization. The left figure represents the path of pesticide loss, and the right figure represents the synergistic mechanism of pesticides. The percentages in the figure (e.g., 100%, 50%, etc.) represent the utilization rate of pesticides.

The fine structure of plant leaves confers a certain degree of hydrophobicity on the leaf surface, so it is difficult for pesticides to attach to them, causing a waste of pesticides (Figure 1). The surface modifiability of nanocarriers allows the attachment of different active groups, which can add or change the charge on pesticides, improving their adhesion to leaves. Compared with traditional pesticides, nanopesticides attach more readily to the leaves and stems of plants, improving their ability to resist rainwater scouring, and thus prolonging the effective period of pesticide contact and improving its utilization rate. For example, the Cry toxin produced by *B. thuringiensis* is easily affected by rain, reducing the period of its activity. However, the application of magnesium hydroxide nanoparticles loaded with Cry1Ac insecticidal protein improved the adhesion of Cry1Ac to cotton leaves by 59%, increasing the death rate of pests by 75%. Cry1Ac is also degraded in acidic environments, after which it exerts no obvious toxicity against cotton and cotton bollworm [102]. Tong et al. used GO and polyamine to build a loading system for oxamycin. This particle has a high loading efficiency for oxamycin, and significantly increases the retention of oxaproxim on plant leaves [103]. Zhao et al. constructed a series of Janus carriers with a cap structure, which improved the deposition and retention of the agent on the leaf surface, and ensured the stable and continuous release of the agent [104].

### 5.4. Nanocarriers Regulate the Release of Pesticides

Nanocarriers can effectively improve the environmental stability of the effective components of pesticides, and can also be used to build a controlled release system that responds to the external pH, redox reactions, enzymes, light, temperature, or other factors. This can reduce the application dose and frequency of pesticides, thus improving their utilization rates [105,106,107]. A nanoconjugate prepared with amino-modified silica loaded with chrysromycin significantly prolonged the effective period of the drug, and the release rate of the effective component was related to temperature, environmental pH, and particle size [108]. Xiang et al. built a pH-responsive drug release system for chlorpyrifos using polydopamine, attapulgite, and calcium alginate, which were self-assembled via hydrogen bonding and electrostatic interactions. This prevented the photolysis of chlorpyrifos and its release in alkaline environments, and the nanopesticide controlled grubs well [65]. Chen et al. prepared a photosensitive slow-release herbicide with biochar, attapulgite, glyphosate, azobenzene, and aminosilicone oil [67]. Under ultraviolet light, the release of glyphosate was controlled by the conversion of azobenzene *trans-cis* and *cis-trans* isomers, in a photosensitive switch. This photosensitive slow-release herbicide is also attached firmly to the weed leaves.

## 6. Safety Evaluation of Nanopesticides and Nanoparticles

The commercialization and popularization of nanopesticides are imperative, but the large-scale application of nanopesticides in the field of agriculture still faces many challenges. Large amounts of nanomaterials are released into the environment, and their presence in the food chain can pose risks to human health [109]. For instance, the large surface areas and other physical and chemical properties of nanoparticles may greatly affect their transformation and bioavailability when they diffuse into the environment. This has aroused widespread concern about their ecological safety. Although there has been extensive research into the safety of nanomaterials, their risk assessment still lags far behind the development and application of this technology.

### 6.1. Toxic Mechanism of Nanomaterials

At present, the toxic mechanisms of nanomaterials in organisms are unclear. Studies have shown that the toxic effects of these materials mainly include the following four factors. First, particles of any nanomaterial can readily penetrate the cell membrane and enter cells, and therefore affect the normal physiological activities of the cell and inhibit photosynthesis [110,111]. Second, some nanomaterials themselves can release toxic ions, such as nano-silver ions, nano-copper ions, and nano-zinc ions [112,113]. The third factor is oxidative damage. Nanomaterials are highly reactive and readily produce reactive oxygen species (ROS). ROS can damage the antioxidant defense system in the mitochondria, produce oxidative stress, and cause functional protein inactivation, and even cell apoptosis, affecting normal physiological functions [17,109,110,111,112,113,114,115,116]. The fourth factor is the biological amplification effect. Nanomaterials can be transferred through the food chain and accumulate in organisms at high trophic levels, in a biomagnification effect [117,118,119].

### 6.2. Study of the Ecotoxicology of Nanopesticides

The particle size of traditional pesticides is large and their effective utilization rates are not high. Only a small proportion of the pesticide actually reaches the target, and most (>60%) is released into the environment during the application process. Nanopesticides have a higher probability of remaining in the environment, and with the increasing application of nanomaterials, they pose a greater risk than other pesticides [120]. Although nanopesticides readily enter harmful organisms, they also pass readily through the tissues and plasma membranes of the human body and nontarget organisms in the environment, entering the cytoplasm or nucleus, with unpredictable consequences. The active components of pesticides and nanomaterials may also exert synergistic toxic effects [121]. The toxic effects of nanopesticides on environmental organisms may be affected by the physical and chemical properties of the pesticides, the exposure route, the environmental behavior of the nanomaterials, and environmental factors. Therefore, ensuring the environmental safety of nanopesticides is much more complex than ensuring that of single nanomaterials or conventional pesticide preparations.

## 7. Advanced Applications and Outlook for Nanopesticides

Nanopesticides can be applied with conventional spraying, root irrigation, or seed coating, consistent with farmers’ traditional practices. In most cases, the application of nanopesticides does not require special spraying equipment. Based on the premise that the preparation of nanopesticides is stable and their quality is assured, there are not many technical barriers to their field application. The development prospects of nanopesticides are positive. In recent years, the following developmental trends have emerged.

(1) RNA nanopesticides have developed rapidly and are expected to be officially registered and used in the next 1–2 years. RNA nanopesticides differ from traditional nanopesticides in that their effective component is double-stranded RNA or small interfering RNA, which targets the key genes of harmful organisms. The establishment and combination of nanodelivery systems and bacterial RNA synthesis technologies are expected to promote the production of RNA nanopesticides [14,15,16,122].

(2) Accelerated research and the development of intelligent nanocarriers will facilitate the development of intelligent agriculture. Intelligent/intelligent nanocarriers allow the precise targeting and efficient delivery of multiple exogenous plant-protection factors through directional transformation. They perform well in terms of manually controlled release, temperature sensitivity, light sensitivity, and magnetically controlled release, and are suitable for application in a variety of environmental scenarios. They are also suitable for future remote-control scenarios on smart farms [113,114,115].

(3) The technology of unmanned aerial vehicles (UAVs) for plant protection has become increasingly mature, and has promoted the application of nanopesticides in the field. At present, most plant-protection UAVs are equipped with small-caliber centrifugal sprinklers. Commonly used traditional pesticides have poor water solubility, which usually causes the blockage and wear of these sprinklers, thus reducing the efficiency of pesticide spraying and limiting the application and popularity of UAV technology for plant protection. The characteristics of nanopesticides make them suitable for plant-protection UAV operations.

## Data Availability

No data were used for the research described in this article.
